# The emerging importance of Shiga toxin-producing *Escherichia coli* other than serogroup O157 in England

**DOI:** 10.1099/jmm.0.001375

**Published:** 2021-07-26

**Authors:** Bhavita Vishram, Claire Jenkins, David R. Greig, Gauri Godbole, Kevin Carroll, Sooria Balasegaram, Lisa Byrne

**Affiliations:** ^1^​ National Infection Service, Public Health England, London, UK; ^2^​ Division of Infection and Immunity, The Roslin Institute and Royal (Dick) School of Veterinary Studies, University of Edinburgh, Easter Bush, EH25 9RG, UK; ^3^​ PHE South East, Surrey and Sussex HPT, Parkside, Chart Way, Horsham RH12 1XA, UK

**Keywords:** epidemiology, haemolytic uraemic syndrome, non-O157 STEC, Shiga-toxin producing *Escherichia coli*

## Abstract

**Introduction:**

Shiga toxin-producing *Escherichia coli* (STEC) can cause severe disease and large outbreaks. In England, the incidence and clinical significance of STEC serogroups other than O157 (non-O157) is unknown due to a testing bias for detection of STEC O157. Since 2013, the implementation of PCR to detect all STEC serogroups by an increasing number of diagnostic laboratories has led to an increase in the detection of non-O157 STEC.

**Hypothesis/Gap statement:**

Due to a bias in testing methodologies to select for STEC serogroup O157 in frontline diagnostic laboratories in most countries, very little surveillance data have been previously generated on non-O157 STEC.

**Aim:**

Five years (2014–2018) of STEC national surveillance data were extracted and descriptive analysis undertaken to assess disease severity of non-O157 STEC strains.

**Methods:**

Data from 1 January 2014 to 31 December 2018 were extracted from the National Enhanced Surveillance System for STEC and analysed.

**Results:**

The implementation of Gastrointestinal Polymerase Chain Reaction (GI-PCR) has resulted in a four-fold increase in the detection of non-O157 STEC cases between 2014 and 2018. There were 2579 cases infected with 97 different non-O157 serogroups. The gender distribution was similar amongst STEC O157 and non-O157 STEC cases with 57 and 56 % of cases being female respectively, but a significantly higher proportion of cases (*P* <0.001) under 5 years of age was observed among STEC O157 (22 %) cases compared to non-O157 STEC (14 %). The most common non-O157 serogroups were O26 (16 %), O146 (11 %), O91 (10 %), O128 (7 %), O103 (5 %) and O117 (3 %). Overall, rates of bloody diarrhoea were highest in O26 (44 %) and O103 (48 %) cases and lowest in STEC O117 cases (17 %). Strains harbouring Shiga toxin *stx1a* caused the highest proportion of diarrhoea (93 %) and caused the same level of bloody diarrhoea as *stx2a* (39 %). However, *stx2a* caused the highest proportion of vomiting (46 %), hospitalisation (49 %) and considerably more HUS (29 %) than other *stx* profiles.

**Conclusion:**

The implementation of PCR targeting *stx* at diagnostic laboratories has shown that non-O157 STEC, most notably STEC O26, are an emerging risk to public health.

## Introduction

Shiga toxin-producing *Escherichia coli* (STEC), also referred to as Verocytotoxin-producing *E. coli* (VTEC) and Enterohaemorrhagic *E. coli* (EHEC), are zoonotic, pathogenic *E. coli*, characterised by the production of Shiga toxin (Stx). STEC are a significant public health concern due to their propensity to cause outbreaks of gastrointestinal disease and haemolytic uraemic syndrome (HUS), a severe life-threatening systemic condition [1]. Ruminants, especially cattle and sheep are the main reservoir for STEC. Transmission occurs through consumption of contaminated food or water or by direct contact with animals or their environment. The STEC pathotype is defined by the presence of the genes encoding Stx type one, type two or both, encoded on a bacteriophage incorporated into the STEC genome. Stx1 and Stx2 can be further divided into subtypes Stx1a-1d and Stx2a-2g; Stx2a is significantly associated with causing severe disease [2].

In England, the most common STEC serogroup is O157 [1]. However, there are more than 400 serogroups other than O157 (non-O157 STEC) and over 100 have been known to cause serious illness and are implicated in causing outbreaks globally. Recent studies have highlighted the importance of emerging non-O157 STEC illness as a cause of diarrhoea, bloody diarrhoea and HUS in humans [3] Although the epidemiology, virulence and clinical significance of STEC O157 are well described in the UK, surveillance data on non-O157 STEC is limited [4, 5]. This is because historically they have been under-reported due to the lack of culture-based methods for the detection of all STEC serogroups.

Detection of STEC O157 in stool specimens relies on its inability to ferment sorbitol, and target organisms appear as colourless colonies on cefixime tellurite-sorbitol MacConkey (CT-SMAC) agar. Most diagnostic laboratories can report presumptive *E. coli* O157 infections within 3 days of specimen collections, enabling a swift public health response. However, most non-O157 STEC do not ferment sorbitol and cannot be differentiated from strains of commensal *E. coli* on CT-SMAC agar. The difficulties associated with detection and primary isolation from faecal specimens hampers public health surveillance, and the true burden of gastrointestinal disease caused by non-O157 STEC remains unclear.

However, since 2013, many diagnostic local hospital laboratories have introduced molecular methods such as commercially available real time Polymerase Chain Reaction (PCR) assays for screening of faecal specimens [1]. PCR gastro-intestinal (GI) panels include primers that detect Shiga toxin genes (*stx 1* and *2*) characteristic of STEC and enable detection of all STEC serogroups. The introduction of *stx* PCR tests by diagnostic laboratories has led to an increase in the number of non-O157 STEC infections detected [1] and consequently an increased number of notifications to local Health Protection Teams (HPT’s).

In response to the increase in the number of non-O157 STEC cases reported, new guidelines were developed in August 2018 for the public health management of STEC O157 and non-O157 STEC cases [4]. The guidance was developed as a public health management tool to prioritise the response to cases most likely to be infected with STEC strains that have the potential to cause HUS. The guidance prioritises public health response to cases infected with STEC harbouring *stx2a*, the primary virulence factor responsible for HUS development, along with the presence of intimin (*eae*) and the age of the host [6, 7].

An epidemiological summary of non-O157 STEC data from England was published by Byrne *et al*. [1] in 2014. Here, we present demographic and clinical data on cases reported from 2014 to 2018. We describe the microbiological characteristics of non-O157 STEC isolated from these cases and describe the clinical outcomes associated with the most common serogroups and virulence profiles to define the emerging threat of non-O157 STEC in England.

## Methods

### Microbiology

Presumptive *E. coli* O157 isolated at diagnostic laboratories from faecal specimens from hospitalised patients and from community cases of gastrointestinal disease are referred to the Gastrointestinal Bacteria Reference Unit (GBRU) for confirmation and typing. Faecal specimens from cases where there is a clinical suspicion of HUS or testing positive for *stx* by PCR are also referred to GBRU where they are cultured for STEC, including non-O157 serogroups. Since April 2015, all isolates of STEC have been further characterised by whole genome sequencing, as previously described [8]. Serum samples were taken from patients with HUS when no STEC was detected in their faecal specimen and were assessed for the presence of antibodies to the lipopolysaccharides of *E. coli* O26, O55, O103, O111, O145 and O157 [9].

### Case definition

A confirmed case of STEC is defined as (i) a case that is culture positive for STEC culture, or PCR positive for *stx* confirmed by GBRU, or (ii) presence of serum antibodies to lipopolysaccharides of *E. coli* O157 or other STEC serogroups, specifically O26, O55, O103, O111, O145, detected at GBRU with clinical symptoms of HUS.

### Data source

Diagnostic laboratories are legally required to notify Health Protection Teams of both clinically suspected cases of HUS and cases where STEC has been detected. An enhanced surveillance questionnaire (ESQ) for STEC is completed for all relevant cases, to obtain a detailed history for the 7 days prior to onset of illness. The STEC operational guidance aims to ensure public health follow-up is focused on the cases with most severe clinical symptoms, and so in general, cases with mild illness (absence of bloody diarrhoea, HUS or hospitalisation) and those in not high risk groups were not followed up with an ESQ [4]. The ESQ collects demographic details; risk status, clinical symptoms, exposures including travel, food and water consumption, and environmental exposures. Completed questionnaires are submitted to the national Gastrointestinal Infections team at PHE (Public Health England) to be included in the National Enhanced STEC Surveillance System (NESSS) where the ESQ data for each patient is linked to microbiological typing data [1].

### Data analyses

Data from 1 January 2014 to 31 December 2018 were extracted from NESSS. Variables for analysis included age group, gender, microbiological typing results (serogroup, *stx* subtypes and *eae*) and clinical symptoms. Cases were categorised into the following age groups based on *a priori* knowledge that children and elderly cases are most frequently reported: 0–5, 6–9, 10–19, 20–39, 40–59, 60–79 and 80 years and over. Clinical symptoms were described only for cases for whom an ESQ was completed and where clinical symptoms were blank on the ESQ, were coded as negative responses.

We describe the demographic, clinical symptoms and microbiological characteristics for cases in England infected with the six non-O157 serogroups most frequently detected. Chi-squared tests were used to compare categorical variables and analyses was performed in Stata v13.

## Results

### Impact of the implementation of PCR at the frontline diagnostic laboratories

**Fig. 1.**
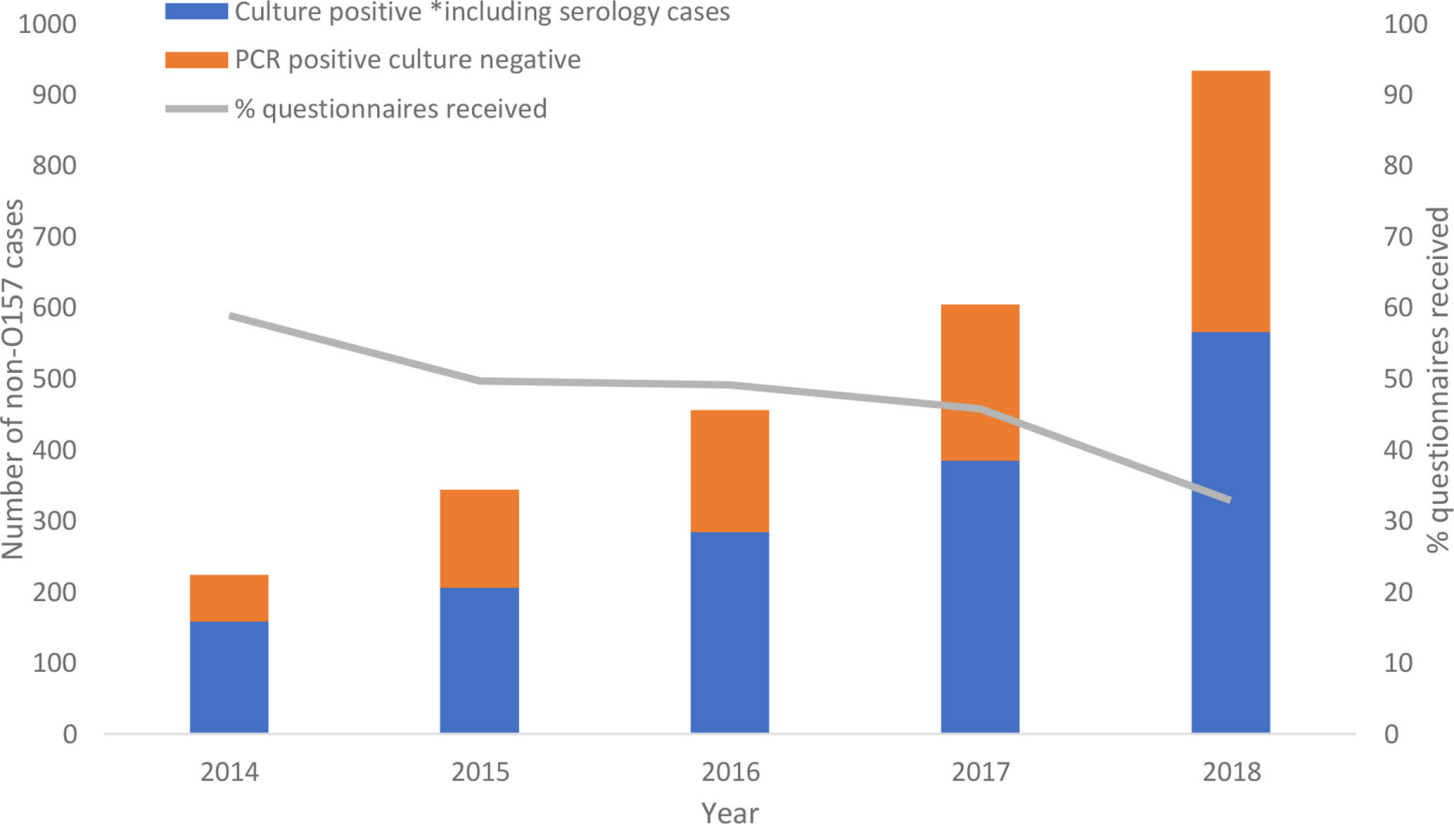
The number of culture positive and PCR positive-culture negative cases non-O157 STEC cases, 2014–2018.

The number of diagnostic laboratories introducing the GI PCR increased from three at the beginning of 2014 to 25 of 117 laboratories (21 %) in England by 2018. This implementation programme resulted in a four-fold increase in the detection of non-O157 STEC with the number of non-O157 STEC cases (culture positive and PCR positive-culture negative) identified each year increasing from 224 cases reported in 2014 to 934 cases reported in 2018 (Fig. 1). The 25 laboratories were nationally distributed, with the majority (*n*=16, 64 %) located in London and the South East of England.

Between 2014 and 2018, 5844 confirmed cases of STEC were reported in England, of these 56 % (*n*=3265/5844) were STEC O157 and 44 % (*n*=2579/5844) were non-O157 STEC. Of those that were identified as non-O157 STEC, 62 % (1599/2579) cases were linked to an STEC isolate (culture positive) and 37 % (964/2579) cases were linked to faecal specimens that were positive by *stx* PCR, but from which STEC were not isolated. Sixteen (21 %) of the 77 HUS cases associated with non-O157 STEC were diagnosed by serology, non-O157 specifically O26 (*n*=8), O55 (*n*=2), O103 (*n*=5) and O111 (*n*=1). The same proportion (21 %, 24/113) of STEC O157 HUS cases were identified by serology.

### Overview of the microbiological data including serotyping and virulence profiles

*Stx* profiles based on the PCR data were available for 99 % (2563/2579) of cases of confirmed non-O157 STEC. Of these 2563 cases, 46 % (1181/2563) carried *stx1* alone, 30 % (768/2563) were *stx1* and *stx2* and 24 % (614/2563) carried *stx2* only. The *eae* gene was detected in 39 % (*n*=996/2563) of the cases.

A total of 97 different serogroups were reported from the 1599 culture positive non-O157 STEC cases. The most common non-O157 serogroups detected were O26 (16 %, 258/1599), followed by O146 (11 %, 180/1599), O91 (10 %, 166/1599), O128 (7 %, 109/1599), O103 (5 %, 85/1599), and O117 (3 %, 54/1599). The complete list of these serogroups detected during this time frame is provided in Table S1 (available in the online version of this article). One hundred and twenty-two isolates did not agglutinate with any of the antisera in the serotyping scheme raised to the known *E. coli* serogroups and were designated ‘O unidentifiable’; including those that did not express the O antigen, and therefore could not be not serotyped.

The *stx* subtype profile was available for 95 % (1519/1599) of the non-O157 isolates from culture positive cases. Nine different *stx* subtype profiles were detected. The most frequently detected *stx* subtypes were s*tx1a* (*n*=445, 29 %) and *stx1c* (*n*=264, 17 %) (Table 1). The most common *stx* subtype profile combination was *stx1c/stx2b* (15 %, 227/1519). The *stx2a* subtype known to be most commonly associated with progression to HUS, was detected in 213/1519 (14 %) isolates, of which 9 % (139/1519) were s*tx2a* only and 4 % (66/1519) were in combination with *stx1a* (Table 1).

**Table 1.** Prevalence of stx subtype combinations reported between STEC non-O157 strains reported between 2014–2018

Stx subtype	N	%
***stx1a***	445	29
***stx1c***	264	17
***stx1c stx2b***	227	15
***stx2b***	145	10
***stx2a***	139	9
***stx1a stx2b***	137	9
***stx1a stx2a***	66	4
***stx2d***	27	2
***stx2e***	15	1
***stx2g***	14	1
***stx2c***	10	1
***stx2 stx1a***	6	0
***stx2a stx2c***	4	0
***stx1a stx2c***	3	0
***stx1a stx2d***	3	0
***stx1c stx2d***	3	0
***stx2f***	3	0
***stx1a stx1c stx2b***	2	0
***stx1a stx2a stx2d***	2	0
***stx1c stx2b stx2c***	2	0
***stx1a stx2a stx2b***	1	0

*denominator=number of non-O157 isolates with whole genome sequencing (1529).

### Overview of the epidemiological data, including age, sex and clinical symptoms

A total of 43 % (1111/2579) questionnaires were received for non-O157 cases between 2014 and 2018 compared to 98 % (3213/3265) of STEC O157 cases. For non-O157 STEC culture positive cases, 64 % (1031/1599) of questionnaires were received compared to 7 % (64/964) questionnaires received for PCR positive, culture negative cases. All questionnaires were received for the 16 serologically confirmed cases. The proportion of non-O157 STEC questionnaires administered from 2014 to 2018 decreased; most notably between 2017 and 2018 with a 28 % reduction in questionnaires (Fig. 1).

Just over half of the total number of all STEC cases, regardless of serogroup, were female (57 %, 3306/5844) and two thirds of cases were over 20 years of age with the highest proportion in the 20–39 age group (27 %, 1577/5844). The gender distribution was similar amongst STEC O157 and non-O157 STEC cases with 57 % (1859/3265) and 56 % (1447/2579) female cases, respectively. With respect to age distribution, a higher proportion of cases was observed in the under 6 years of age group among STEC O157 cases compared to non-O157 STEC cases (22 % vs 14 %, *P* <0.001). Among the HUS cases, 82 % (63/77) were children.

Overall higher proportions of STEC O157 cases had clinical symptoms compared with non-O157 cases; diarrhoea (91 % vs 84 %, *P* <0.001), bloody diarrhoea (59 % vs 32 %, *P* <0.001), fever (34 % vs 30 %, *P*=0.016), abdominal pain (80 % vs 69 %, *P* <0.001) and vomiting (34 % vs 29 %, *P*=0.005). A higher proportion of STEC O157 cases were admitted to hospital for illness (32 % vs 21 %, *P* <0.001) although more non-O157 cases developed HUS than O157 cases (7 % vs 4 %, *P* <0.001) (Table 2).

**Table 2.** Disease severity among non-O157 cases for whom questionnaires were completed in England, 2014–2018

Serogroup	No. of ESQs	Diarrhoea		Bloody diarrhoea		Fever		Abdominal pain		Vomiting		Admitted to hospital		HUS	
		N	%	N	%	N	%	N	%	N	%	N	%	N	%
**O157**	3213	2930	91	1907	59	1078	34	2572	80	1080	34	1035	32	113	4
**Non-O157**	1111	930	84	356	32	329	30	766	69	323	29	234	21	77	7
**O26**	194	176	91	86	44	54	28	143	74	71	37	55	28	19*	10
**O146**	117	94	80	22	19	32	27	71	61	25	21	14	12	0	0
**O91**	112	85	76	27	24	36	32	73	65	31	28	10	9	0	0
**O128**	66	52	79	14	21	21	32	44	67	10	15	8	12	0	0
**O103**	50	44	88	24	48	13	26	35	70	14	28	13	26	6†	12
**O117**	23	19	83	4	17	3	13	16	70	4	17	2	9	0	0

*9/19 STEC O26 HUS cases had *stx* subtype, six were *stx2a* and three were *stx1a* and *stx2a*, the other 10 cases were determined by serology.

†1/6 STEC O103 HUS cases had *stx1a*, the other five cases were identified by serology.

The frequency of clinical symptoms in the most common *stx* profiles possessed by non-O157 STEC were analysed (Table 3). *Stx1a* was associated with the highest frequency of diarrhoea (93 %) and with the same frequency of bloody diarrhoea as *stx2a* (39 %). The *stx1a, stx2a* profile was associated with bloody diarrhoea (49 %) and abdominal pain (84 %). However, the *stx2a* profile was associated with vomiting (46 %), hospitalisation (49 %) and a considerably higher frequency of HUS (29 %) than other *stx* profiles. Other *stx* subtypes that were also associated with HUS included *stx1a* (1 %) and *stx1a, stx2a* (5 %).

**Table 3.** Prevalence of *stx* subtype combinations amongst STEC O157 and the top six non-O157 serogroups, 2014–2018

Stx subtype	N	%
**O157**		
*stx1a*	663	29
*stx1a stx2a*	582	25
*stx1a stx2a stx2c*	507	22
*stx1a stx2c*	458	20
*stx1c stx2b*	31	1
*stx2a*	30	1
*stx2a stx2c*	20	1
*stx2b*	1	0
*stx2c*	1	0
*stx2d*	1	0
Total	2294	
**O26**		
*stx1a*	138	58
*stx1a stx2a*	53	22
*stx2a*	40	17
*stx2 stx1a*	5	2
*stx1a stx2a stx2d*	2	1
*stx1a stx2c*	1	0
Total	239	
**O146**		
*stx1c*	77	46
*stx1c stx2b*	60	36
*stx2b*	28	17
*stx1c stx2d*	3	2
*stx1a*	1	1
Total	169	
**O91**		
*stx1a stx2b*	125	78
*stx2b*	27	17
*stx1a*	3	2
*stx1c stx2b*	2	1
*stx1a stx1c stx2b*	1	1
*stx1a stx2a stx2b*	1	1
*stx1c*	1	1
Total	160	
**O128**		
*stx1c stx2b*	68	64
*stx2b*	27	25
*stx1c*	9	8
*stx1a*	2	2
*stx2d*	1	1
Total	107	
**O103**		
*stx1a*	79	99
*stx2a*	1	1
Total	80	
**O117**		
*stx1a*	50	96
*stx1a stx2b*	1	2
*stx2b*	1	2
Total	52	

### Demographic comparisons for the most common serogroups: O26, O146, O91, O128, O103, O117

**Fig. 2.**
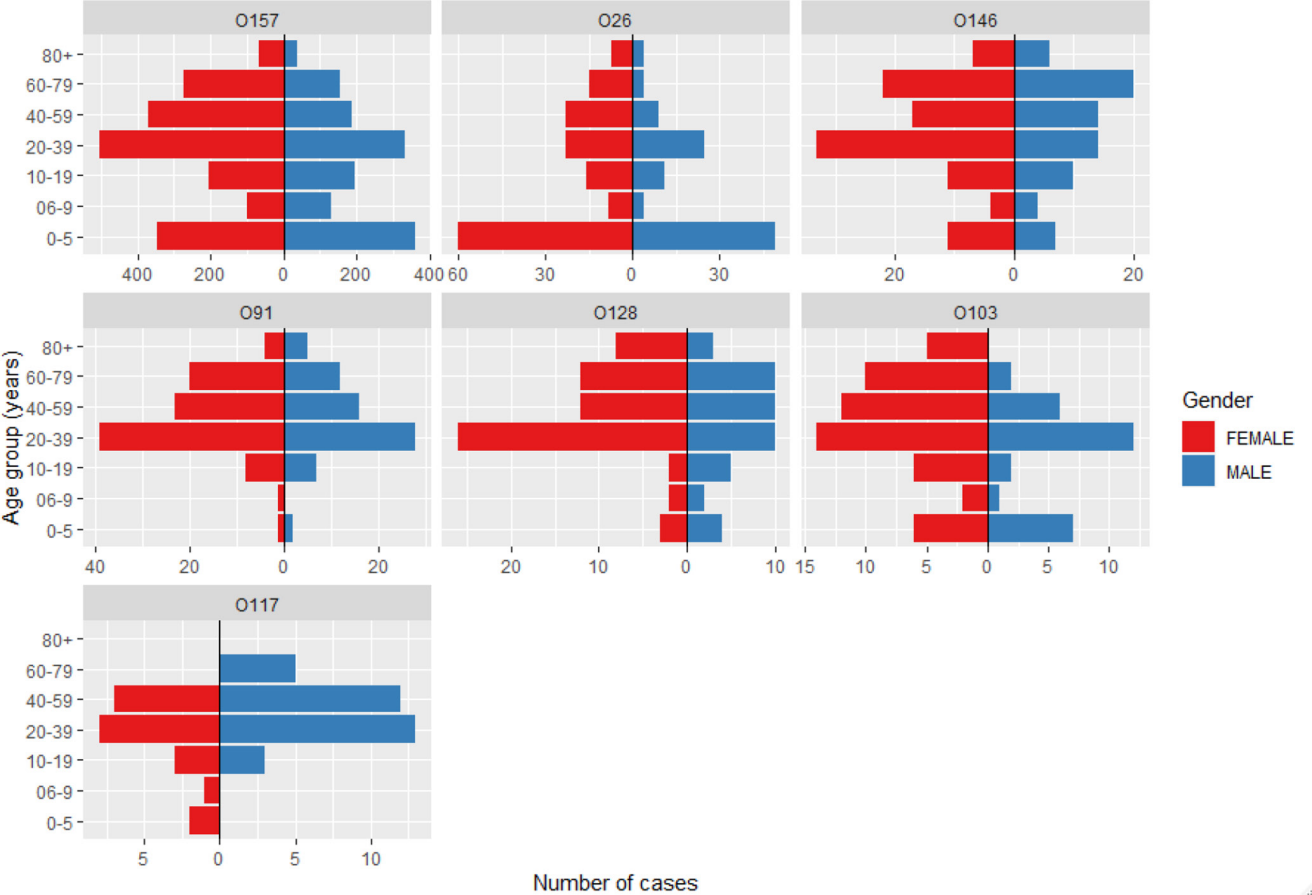
Age-sex distribution of STEC serogroups: O157, O26, O103, O91, O128, O146, and O117.

The age and sex distribution of cases of STEC O26 was similar to that of STEC O157 cases with the highest number of cases reported in children under 6 years of age amongst both serogroups. However, there was a higher proportion of children under six (42 %, 109/258) amongst the cases of STEC O26 compared to O157 cases (22 %, 709/3265), (42 % vs 22 %, *P* <0.001). Cases infected with STEC serogroups O91, O128, O103 and O146 serogroups had higher proportion of cases in adults (> 20 years of age); STEC O91 (89 %, 147/166), STEC O128 (84 %, 91/109), STEC O103 (68 %, 41/60) and STEC O146 (74 %, 133/180). STEC O117 was the only serogroup where cases were highest in males (61 %, 33/54), particularly in 20–39 (57 %, 12/21) and 40–59 (63 %, 12/19) age group (Fig. 2).

### Virulence gene profiles and clinical outcome comparisons for the most common serogroups: O26, O146, O91, O138, O103, O117

The *stx1a* subtype alone was the most frequent subtype found in STEC O26 (58 %, 138/239), STEC O103 (99 %, 79/80), and STEC O117 (96 %, 50/52) isolates (Table 3). Amongst the isolates of STEC serogroups O146, O91 and O128 the most common *stx* subtype profiles were *stx1c* only (46 %, 77/169), *stx1a/stx2b* (78 %, 125/160) and *stx1c/stx2b* (64 %, 68/107), respectively. All isolates belonging to STEC serogroups O26 and O103 isolates possessed *eae*, whereas STEC serogroups O91, O117, O128, and O146 did not. A table of the microbiological characteristics of the top six non-O157 serogroups are provided in Table S2.

The same proportion of STEC O26 and STEC O157 cases reported diarrhoea (91 % vs 91 %, *P*=0.823). The highest frequency of bloody diarrhoea was reported by patients infected with STEC O157 (59 %) compared to O103 (48 %, *P*=0.537) and O26 (44 %, *P* <0.001). Frequency of vomiting was similar in STEC O26 and STEC O157 cases (37 % vs 34 %, *P*=0.393). Frequency of hospitalisations were comparable with those for STEC O157 amongst cases with STEC O26 (28 % vs 32 %, *P*=0.263) and STEC O103 (26 % vs 32 %, *P*=0.601). The proportion of cases that developed HUS were higher in those infected with STEC O26 (10 %, *P* <0.001) and STEC O103 (12 %, *P*=0.007) cases, than for cases of STEC O157 (Table 4). Of the 25 cases with HUS, ten had *stx* subtype data available, the others were confirmed by serological diagnosis. Among the HUS cases associated with STEC O26, six possessed the *stx2a* subtype and three the *stx1a, stx2a* subtype profile. Of the STEC O103 HUS cases, *stx* subtype was only available for one isolate (*stx1a*).

**Table 4.** Disease severity among the top seven most common STEC non-O157 *stx* profiles combinations, 2014–2018

Stx subtype	No. of ESQs	Diarrhoea		Blood stools		Abdominal pain		Fever		Vomiting		Admitted to hospital		HUS	
	N	N	%	N	%	N	%	N	%	N	%	N	%	N	%
***stx1a***	241	223	93	93	39	189	78	73	30	63	26	38	16	2	1
***stx1c***	126	97	77	27	21	83	66	34	27	29	23	16	13	0	0
***stx1c stx2b***	143	117	82	27	19	88	62	40	28	26	18	19	13	0	0
***stx2b***	95	72	76	16	17	64	67	29	31	17	18	12	13	0	0
***stx2a***	133	105	79	52	39	87	65	36	27	61	46	49	37	29	22
***stx1a stx2b***	96	71	74	19	20	63	66	30	31	27	28	8	8	0	0
***stx1a stx2a***	61	54	89	30	49	51	84	16	26	17	28	11	18	3	5

## Discussion

Over the last four decades, surveillance of STEC in England has focused on STEC O157 as the serogroup perceived to be of most concern to public health. The recent implementation of PCR targeting *stx* at diagnostic laboratories has highlighted that other serogroups are also causing gastrointestinal disease in England and are often associated with severe clinical outcomes. A combination of the year on year increase in the number of laboratories implementing PCR and the lack of uniform coverage across the country means that it was not possible to assess trend data from this dataset. However, by analysing the pathogenicity profiles of the isolates and the clinical outcomes of the patients, we were able to assess the risk to public health.

Between 2014–2018, the most common six non-O157 STEC serogroups in England were O26, O146, O91, O128, O103 and O117, compared to the equivalent top six in the United States of O26, O103, O111, O121, O145 and O45 [10]. Data from the United States showed that 75–80 % of the reported top six non-O157 STEC serogroups isolated are from humans with severe symptoms including bloody diarrhoea and HUS [11, 12]. Data from Europe indicates that the top four non-O157 serogroups in Europe are O26, O103, O91 and O145 [13]. In Ireland, the most common serogroup was O26 followed by O157, O145, O103, O5 and O111 [14]. STEC O26 was the most common serogroup associated with HUS in Ireland in 2017. STEC O26 and O103 were in the top six non-O157 serogroups in all three countries as well as amongst European Union/European Economic Area countries [13], and were associated with greater disease severity (HUS and hospitalisation) in our dataset.

In countries such as Ireland and Japan, using a more comprehensive approach to the detection of STEC serogroups through the use of PCR, STEC O26 is often associated with a higher burden of gastrointestinal disease than STEC O157 [15–17]. In this study, STEC O26 was the most common non-O157 STEC serogroup and the serogroup most similar to STEC O157 with respect to disease severity and the age and sex profile of the cases. Like STEC O157, a relatively high proportion of STEC O26 harbour *stx2a* and *eae*, the combination of virulence factors most likely to be associated with severe symptoms including bloody diarrhoea, and progression to HUS [2]. The number of cases, the association with HUS and the evidence that children are commonly infected, is clear evidence that STEC O26 should be considered a similar risk to public health as STEC O157.

Although less common, and in this dataset harbouring *stx1a* only, cases infected with STEC O103 also exhibit poor clinical outcomes. The association between *stx1a* and severe bloody diarrhoea and hospitalisation has been previously described [2, 5]. In our study, STEC O103 was associated with a higher proportion of HUS cases than STEC O157. However, all but one of these diagnoses were made using a sero-diagnostic assay that detects antibodies to the lipopolysaccharide of *E. coli* O103, rather than to the Shiga toxin. Cross reactions with other *E. coli* serogroups and other gastrointestinal pathogens have been described for these serum assays and therefore these results should be interpreted with caution [18]. PCR targeting *stx* followed by culture for STEC is the recommended approach rather than serodiagnosis [19].

Despite harbouring *stx1a*, the proportion of cases of STEC O117 reporting bloody diarrhoea, vomiting and hospitalisation were lower when compared to cases infected with other serogroups, including STEC O103 which also possess *stx1a*. The absence of *eae*, a gene that encodes proteins involved in the mechanisms of attachment of STEC to the gut mucosa of the host, may be a factor in cases of STEC O117 reporting less severe clinical outcomes. Despite the absence of *eae*, patients infected with STEC O117 have been known to shed for long periods after recovery, indicating that other mechanisms of attachment to the gut are at play [20, 21]. Previously, cases of STEC O117 have been linked to sexual transmission among men who have sex with men (MSM), and this exposure is the likely explanation for the high male to female ratio in adults observed for this serogroup [22, 23]. Long-term shedding in asymptomatic individuals is of particular concern with respect to facilitating transmission of gastrointestinal pathogens in the MSM community [24]. This serogroup is also known to be a cause of travellers’ diarrhoea to destinations regarded as high-risk regions, such as Latin America, Africa and the Indian sub-continent [20, 21]. Previous studies have shown that this STEC serogroup exhibits higher levels of antibiotic resistance than most other STEC serogroups [25]. As antibiotics are often used to treat travellers’ diarrhoea, and gastrointestinal symptoms in MSM, multidrug resistance in this STEC serogroup is an additional public health concern. The other common STEC serotypes exhibit lower levels of antimicrobial resistance [26].

Due to current laboratory practices in England, the available data on non-O157 STEC serogroups may be biassed as faecal specimens from patients with most severe symptoms, such as HUS, are more likely to be referred to GBRU, and to have questionnaires undertaken. Another limitation to this study, was that we were unable to estimate a denominator to calculate incidence by serogroup, age-sex and geography as details of contract arrangements for referral of samples from primary care and catchment areas of each diagnostic laboratory are not known. Although, we were not able to provide a detailed summary on the clinical outcomes of all serotypes in this study, it is important to highlight the clinically significant STEC O55:H7 [17, 18]. This strain emerged in South West of England in 2014, continued to cause small, geographically discreet outbreaks until 2018, and was associated with a high proportion (~50 %) of cases that developed HUS (Sawyer *et al*., submitted).

Previous studies have compared non-O157 STEC as a group to STEC O157 [9–11]. This is the first study where we have accumulated sufficient data to compare clinical, demographic and microbiological data between different non-O157 serogroups in England. We have shown that the comparing STEC non-O157 as a group to O157 is not informative, and risk assessment should be based on Stx subtype and genes involved in attachment to the gut mucosa. Our study provides further evidence that the virulence gene profile *stx2* and *eae* are risk factors for the development of STEC-HUS [26–28]. However, there is increasing evidence that the presence of *stx1a* is also a risk factor for severe symptoms and caused the highest proportion of diarrhoea (93 %) and the same proportion of bloody diarrhoea (39 %) as *stx2a*. In the light of the evidence presented here, and elsewhere, we recommend that *stx1a* in combination with *eae* be included as a potential higher risk profile [5].

Moving forward, widespread implementation of the molecular platforms in diagnostic laboratories and subsequent follow up via NESSS will enhance our ability to determine the true incidence of non-O157 STEC infection on England, the burden in terms of morbidity and mortality, and whether there are niche risk exposures for particular strains.

## Supplementary Data

Supplementary material 1
